# Prader-Willi syndrome, excessive daytime sleepiness, and narcoleptic symptoms: a case report

**DOI:** 10.1186/1752-1947-8-127

**Published:** 2014-04-17

**Authors:** Sara V Weselake, Jessica L Foulds, Robert Couch, Manisha B Witmans, Daniela Rubin, Andrea M Haqq

**Affiliations:** 1 Department of Medicine, University of Manitoba, 727 McDermot Avenue, Winnipeg, MB R3E 3P5, Canada; 2 Department of Pediatrics, University of Alberta, 1C4.09 Walter C. Mackenzie Centre, 8440-112 Street NW, Edmonton, AB T6G 2R7, Canada; 3 Department of Kinesiology, California State University, 800 North State College Boulevard, Fullerton, CA 92831, USA

**Keywords:** Pediatrics, Prader-Willi syndrome, Modafinil, Excessive daytime sleepiness, Narcolepsy, Cataplexy

## Abstract

**Introduction:**

Sleep abnormalities, including narcolepsy and cataplexy, are a common feature of Prader-Willi syndrome. Long-term treatment with the central nervous system stimulant modafinil has not been reported. In this case report we present a longitudinal perspective of sleep abnormalities in a nine-year-old Caucasian girl with Prader-Willi syndrome from age two to age nine, and detail the response to treatment with the central nervous system stimulant modafinil.

**Case presentation:**

Our patient presented at two years of age with hypersomnia and narcoleptic episodes with cataplectic features. Initial polysomnograph testing revealed adequate sleep efficiency, but increased sleep fragmentation especially during rapid eye movement sleep. The narcoleptic episodes continued and a repeat polysomnograph at age five years confirmed features consistent with narcolepsy. Further sleep studies at six years, including a multiple sleep latency test, demonstrated signs of excessive daytime sleepiness. Treatment with modafinil was initiated at age seven years six months due to persistent hypersomnia and narcoleptic symptoms. Two polysomnograph studies were performed following treatment with modafinil, at age eight years six months and nine years three months. These studies showed excellent sleep efficiency and improvement of rapid eye movement sleep parameters, supporting the beneficial effects of long-term modafinil therapy.

**Conclusions:**

Long-term modafinil therapy may ameliorate the sleep disturbances of Prader-Willi syndrome and should be the focus of future clinical trials.

## Introduction

Prader-Willi syndrome (PWS) is a genetic disorder occurring in 1/10,000 to 25,000 live births. PWS is associated with infantile hypotonia and failure to thrive, followed by childhood-onset hyperphagia and progressive obesity, short stature, hypogonadism, behavioral issues, delayed cognition and sleep disturbances [1]. Sleep abnormalities are a common symptom of PWS, most often presenting as excessive daytime sleepiness (EDS) [2,3]. Abnormalities in sleep physiology have been reported including: reduced percentage of rapid eye movement (REM) sleep and decreased non-rapid eye movement (NREM) sleep instability, decreased REM sleep latency or sleep-onset REM (SOREM), ventilatory dysfunction, and obstructive sleep apnea [4]. Additionally, there have been a number of studies reporting narcoleptic-like symptoms such as sleep attacks, cataplexy or a transient loss of muscle tone, sleep paralysis and hypnagogic hallucinations. Modafinil, a central nervous system (CNS) stimulant of postsynaptic alpha-1 adrenergic receptors, is currently used in the treatment of narcolepsy and idiopathic hypersomnia, but has not been well studied in the treatment of sleep abnormalities in PWS [5]. We report a case of a nine-year-old PWS girl with cataplexy and narcolepsy first noted in infancy and chronicle the sleep abnormalities over seven years, and the effect of modafinil on these sleep abnormalities over three years of therapy. This provides a previously unreported longitudinal perspective of the impact of modafinil on the quality of life of patients with PWS and EDS. Further, this report also briefly examines the neurobiologic basis of disordered sleep in PWS.

## Case presentation

This nine-year-old Caucasian girl was born at 41 weeks’ gestation via induced vaginal delivery, and weighed 3260g. She was hypotonic and lethargic at birth with failure to thrive and was nasogastric tube-fed initially for one month. At 15 months of age, she was diagnosed with PWS with a rare imprinting center methylation mutation. She has physical features characteristic of PWS including almond-shaped eyes, down-turning corners of the mouth, small hands and a prominent metopic suture. Other features of PWS include gross motor delay, social delay, obsessive-compulsive behavior and rigid thinking. Her family history is notable for a number of members (including our patient’s father) who suffer from EDS. None of these individuals, however, report characteristics of narcolepsy such as cataplexy, hypnagogic hallucinations, or sleep paralysis.

Our patient was referred to a pediatric pulmonologist at age two years 10 months for a screening polysomnograph (PSG) sleep study prior to initiation of growth hormone (GH) therapy. An initial screening overnight oximetry study was carried out before the PSG due to concerns that she might not tolerate the monitoring equipment. The screening oximetry was normal; no significant desaturations to suggest obstructive sleep apnea were found. At the follow-up with the sleep specialist, our patient’s mother reported episodes suggestive of narcolepsy. Dating back to early infancy, episodes were reported of our patient falling forward for a few seconds at a time with her eyes rolling back in her head. During these events, she was unresponsive, but appeared to be conscious. The events occurred three to four times per week and were provoked by laughter. The episodes typically occurred after waking from a nap in the mornings, and resolved spontaneously. She was not sleepy afterwards. The episodes were determined not to be seizures; no other associated motor activity, eye deviation or stereotypy consistent with a description of a seizure was present. Daytime hypersomnia was also noted with sleep duration of greater than 12 hours per night in addition to a two- to three-hour daytime nap.

The initial sleep study revealed adequate sleep efficiency, but disrupted sleep overall (Table 1). Specifically, our patient did not cycle through all stages of sleep as expected; sleep fragmentation was noted, especially during the REM phase. Mild sleep-disordered breathing (SDB) was identified, consisting of four central apneas, one obstructive apnea, and three mixed apneas, particularly within REM sleep. Two obstructive, but no central, hypopneas were observed. The apnea-hypopnea index (AHI) was 1.4 events per hour. The average end-tidal carbon dioxide (EtCO_2_) measurement was 45mmHg (maximum of 61mmHg). The average oxygen saturation (SpO_2_) was 95 percent (minimum of 78.7 percent).

**Table 1:** **Polysomnograph results in a patient with Prader-Willi syndrome and narcoleptic and cataplectic symptoms**^**1**^

	**2y 10mo**	**5y 3mo**	**6y 10mo**	**8y 5mo**	**9y 3mo**
Sleep architecture					
Total sleep time (min)	420.5	489.5	516.5	520	492
Sleep efficiency (%)	84.9	90.8	92.7	94.4	95.1
Sleep stages total sleep time					
Stage 1 (%)	6.5	2.2	2.5	0.6	2.1
Stage 2 (%)	43.4	48.1	57.9	64.7	59.3
Stage 3 and 4 (%)	22.5	25.8	18.6	18	18.5
REM (%)	27.6	23.9	21.0	16.8	20.1
Apnea events					
Central apnea index (events per hour)	0.57	0	0.46	2.08	2.32
Obstructive apnea index (events per hour)	0.14	0.12	0	0.12	0
Number of mixed events per hour	0.43	0	0.58	0	0
Hypopnea events (events per hour)	0.29	0.25	0.23	0.34	3.05
Apnea-hypopnea index (events per hour)	1.4	0.4	1.3	2.7	5.6
End-tidal carbon dioxide (ETCO_2_)					
Mean total recorded ETCO_2_ (mmHg)	44.5	46	42	43	45
Maximum total recorded ETCO_2_ (mmHg)	60.9	60	51	50	54
Oxygen saturation (SaO_2_)					
Mean total recorded SaO_2_ (%)	94.1	95	97	94	96
Minimum total recorded SaO_2_ (%)	78.7	87	88	84	84
Anthropometrics					
Height (cm)	84	97	104	110	116
Weight (kg)	11.25	15.2	17.4	20.0	21.4
BMI- z score	0.12	0.66	0.39	0.25	−0.23

^1^Modafinil therapy was initiated at age seven years six months; growth hormone therapy was initiated at age eight years eight months. REM, rapid eye movement; BMI body mass index.

A repeat overnight PSG was completed at five years three months of age due to ongoing concerns of EDS and cataplectic episodes. The goals for treatment were to optimize school performance and ensure our patient’s safety. At that time, episodes of cataplexy were still occurring, mainly in the mornings upon awakening. The results of the second PSG study (Table 1) were abnormal and suggestive of narcolepsy. Spikes were noted on electroencephalography without any seizure activity and a shortened REM sleep latency was observed. The overall degree of SDB was improved compared to the previous measurement. Abnormal respiratory events were limited to one obstructive apnea and two obstructive hypopneas with an AHI of 0.4 events per hour. The average EtCO_2_ was 46mmHg; the SpO_2_ was also improved with an average of 95 percent (minimum of 87 percent).

Another follow-up PSG was done at age six years and 10 months. This PSG again showed excellent sleep efficiency and decreased REM sleep latency (Table 1). However, worsening sleep disruptions were observed, with increased spontaneous arousals. SDB or obstructive sleep apnea in REM sleep (an AHI of 4.4 events per hour) were also worse than previous observations. Finally, a multiple sleep latency test (MSLT) was performed to objectively quantify the degree of sleepiness (Table 2). This test consisted of five nap opportunities, given at two-hour intervals. Her MSLT results showed the following sleep latencies on five respective naps: 9min 18sec; 1min 44sec; 54sec; 6min 23sec; 3min 55sec. The overall mean sleep latency was 4min 27sec. Two SOREM periods were also noted.

**Table 2:** Multiple sleep latency test results in a patient aged six years 10 months with Prader-Willi syndrome and narcoleptic and cataplectic symptoms

**Nap opportunity**	**Sleep onset latency**	**Sleep onset REM**
1	9 minutes, 18 seconds	Yes
2	1 minute, 44 seconds	No
3	54 seconds	No
4	6 minutes, 23 seconds	No
5	3 minutes, 54 seconds	Yes
Mean sleep latency	4 minutes, 27 seconds	

REM, rapid eye movement.

The results of the above sleep studies combined with the report of narcoleptic and cataplectic episodes led to the initiation of treatment at age seven years six months with a CNS stimulant, modafinil. The initial dose was 50mg in the mornings and scheduled naps were presented throughout the day. Two months later, the dose of modafinil was increased to 100mg daily. With modafinil treatment, episodes of narcolepsy and cataplexy were not entirely eliminated, but much improved. A decreased level of EDS and fewer cataplectic episodes were observed post-therapy and safety was no longer a concern. Our patient had no history of allergy and no adverse effects of modafinil were reported.

After a year of modafinil therapy a PSG was completed, at age eight years and six months. This study demonstrated improvement compared to all previous tests, showing excellent sleep efficiency (Table 1). However, sleep latency remained short at 6 minutes and 57 seconds. The REM sleep latency was within normal limits (1 hour) in contrast to previously abnormal results. Disruption of sleep was also observed, with increased time spent in stage 2 sleep (67.4 percent of total sleep time; TST) and less time in REM sleep (16.8 percent of TST). Additionally, SDB was demonstrated (mostly central apneas with an AHI of 2.7 events per hour). These apneas were particularly prominent in REM sleep; the REM AHI was 10.8 events per hour and a lower baseline SpO_2_ of 93 percent and an EtCO_2_ in the mid-40s was observed. Finally, there was a slightly elevated periodic limb movement index of 6.6 events per hour.

At age eight years and eight months, our patient was started on GH and a PSG test was recommended six months following GH initiation. The dose of modafinil was increased to 150mg per day to improve the narcolepsy and cataplectic episodes. This fifth PSG was done at age nine years and three months. Again, excellent sleep efficiency was noted. The REM sleep latency was slightly prolonged again at 1 hour 21 minutes, and the proportion of REM sleep was less than expected (20.1 percent of TST). Overall, excellent sleep efficiency was noted in all stages of sleep despite diminished REM sleep and a slightly prolonged sleep latency. Moderate SDB was again demonstrated (mostly central apneas with an AHI of 5.6 events per hour and a REM AHI of 17.6 events per hour). Periodic limb movements again occurred, with three events per hour. GH therapy elicited several benefits in our patient, including increased height and growth rate, increased proportional hand and foot sizes, a decrease in body fat and an increase in lean body mass and increased physical performance. Other potential benefits of the GH therapy were not measured, such as resting energy expenditure, serum cholesterol and bone mineral density.

Long-term modafinil therapy resulted in objective improvement in her PSG results, in conjunction with clinical improvement in her mood and a decreased frequency of naps. An increased level of alertness and stamina throughout the day was noted by caregivers. Without modafinil use, our patient became tired and irritable by early afternoon. With modafinil therapy, our patient no longer required naps after school and was more social, alert, and less irritable. A decrease in the cataplectic episodes was demonstrated; the remaining episodes were limited to the morning hours prior to modafinil administration.

## Discussion

Sleep abnormalities in patients with PWS lead to detrimental effects on daily functioning and learning. Our report provides a unique longitudinal perspective on the disordered sleep of a PWS patient, and the effectiveness of long-term modafinil use for the treatment of EDS in a young girl with PWS over the course of three years. EDS is a common symptom of PWS, affecting between 70 and 85 percent of PWS individuals [4]. Caregivers often report increased sleepiness in both children and adults with PWS; individuals with PWS also self-report higher levels of EDS compared to other intellectually disabled groups [6]. EDS is also objectively demonstrated through abnormal MSLT results in PWS patients. A collection of studies reviewed by Maas *et al.* reported severe sleepiness (MSLT score less than 5 minutes) in 40 to 50 percent of adults with PWS and moderate to severe sleepiness (MSLT score less than 10 minutes) in 70 to 100 percent [2,7]. A combination of obesity, craniofacial abnormalities, sleep apnea and abnormalities of sleep structure are proposed contributing factors related to their daytime hypersomnia [4]. Sleep difficulties can functionally impair attention and behavior and overall cognitive status and school functioning [1]. Our patient was initially noted to nap for two to three hours each day and sleep for 12 hours during the night. These sleep periods are relatively long compared to age-matched peers; the average sleep duration for eight-year-olds is 10.6 ± 0.6h per 24h period [8]. A MSLT performed at age six revealed severe pathological sleepiness, with a MSLT score of less than 5 minutes. Generally, prepubertal children have mean sleep latencies greater than 15 minutes [8]. Additionally, the two SOREMs found on our patient’s MSLT are typical features of both narcolepsy and PWS [4].

A variety of nighttime sleep architecture disturbances are also reported in PWS. The most commonly observed dysfunction is a decreased REM sleep latency and the presence of SOREM periods [9]. Other abnormalities include reduced percentage of REM sleep, fragmented REM sleep, and decreased NREM sleep instability [4]. Studies specific to pediatric PWS patients show a tendency for increased stage I sleep and decreased REM latency. The PSG testing over the course of six years in our patient illustrates many of these findings. Our patient also had a family history of EDS, which may contribute to her significant sleep abnormalities.

Modafinil is a psychostimulant prescribed for the treatment of sleepiness in narcolepsy and other hypersomnia disorders. The mechanism or set of mechanisms through which modafinil promotes arousal and activity are incompletely understood; however, it is believed to activate the catecholamine systems at α and β adrenergic receptors and dopamine receptors [10]. Treatment with modafinil in small pilot studies has been shown to improve the sleepiness in patients with PWS [11] and was clinically beneficial for our patient. The drug, however, has no reported benefit on control of cataplexy [12]. Our patient began treatment with modafinil at age seven years six months of age (initially at 50mg daily and then progressed to 150mg daily). Two PSGs were performed subsequent to the initiation of this drug and both revealed an improvement in a number of sleep parameters. The PSG performed at ages eight and nine demonstrated excellent improved sleep efficiencies of 94 and 95 percent, respectively. Additionally, modafinil therapy in our patient eliminated SOREM periods and decreased REM sleep latency. However, a reduction in REM sleep remained at the final PSG at eight years of age, indicating that not all aspects of sleep abnormality were ameliorated with modafinil treatment. It is worth noting that at the increased modafinil dosage (150mg daily), a longer period of REM sleep was observed. Therefore, further optimization of modafinil dosing might be helpful. A MSLT would have been useful in objectively assessing the impact of modafinil on our patient’s hypersomnia after a period of therapy; however, the patient refused further MSLT testing. A significant improvement in alertness, irritability, and reduction in naps and cataplectic episodes was noted by caregivers.

The neurobiologic basis of sleep abnormalities in PWS are unclear and likely multifactorial. One of the proposed causes of EDS in PWS is a combination of obesity and sleep apnea and its disruptive effect on the quality of sleep. However, resolution of the above issues in PWS does not completely eliminate their sleep abnormalities; our subject was not obese [4]. Therefore additional mechanisms for their EDS may include hypothalamic dysfunction (specifically hypothalamic control of circadian rhythm and NREM/REM cycling) [10].

**Figure 1:**
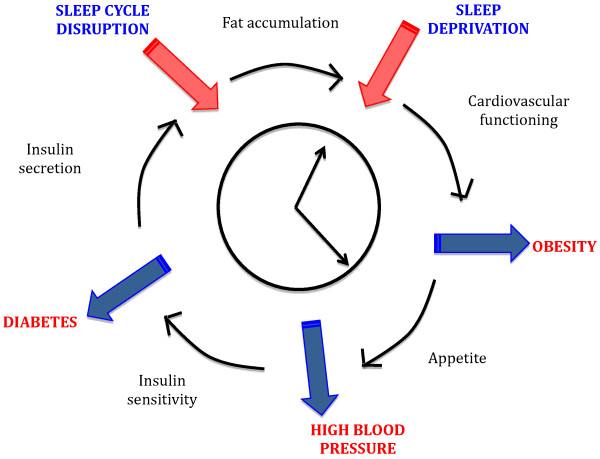
Sleep disturbances and metabolic functioning.

Further understanding of how genes in the PWS locus are connected to sleep functioning offers insight into an underlying cause of the sleep abnormalities observed in PWS. For example, one of the genes linked to the phenotype of PWS is *MAGEL2* which is expressed in the suprachiasmatic nucleus (SCN) [13], the location of the body’s core circadian clock. These findings in the mouse model of PWS suggest a possible connection between the genetics of the PWS, disrupted circadian rhythm and the observed sleep abnormalities. A disrupted circadian rhythm associated with PWS might also manifest as specific clinical metabolic (central obesity, type 2 diabetes, and cardiovascular disease) and sleep abnormalities observed in this condition. Research has also illustrated the contribution that altered circadian rhythm and sleep patterns might result in disrupted metabolic functioning, including impaired insulin sensitivity and increased risk of diabetes, obesity and cardiovascular events in the general population (Figure 1) [14]. Therefore, it is important to treat sleep issues in patients with PWS not only for improvement of sleep and energy levels, but also to prevent further metabolic deterioration in this disease. While one open-label study of modafinil treatment has been carried out in PWS patients, duration of treatment was relatively short (1.7 to 14.7 months), no placebo was employed and a small number of subjects were included (n = 9) [11]. The long-term benefit of modafinil for EDS and SDB associated with PWS should be further explored in larger, controlled studies that also address metabolic outcomes.

## Conclusions

There are a number of published case reports of pediatric patients with Prader-Willi syndrome, cataplexy and narcolepsy. This report provides novel information regarding the long-term improvement of both sleep parameters and narcolepsy/cataplexy post-modafinil therapy. In our patient, EDS impaired nighttime sleep and narcoleptic/cataplectic episodes resulted in significant decrease in overall quality of life. Treatment with modafinil significantly improved these symptoms and improved the quality of life of our patient over the course of several years. Given the high incidence of SDB and sleep disturbances in PWS, it is imperative to routinely screen for sleep and breathing problems during clinic visits and work in conjunction with a pediatric sleep specialist to manage these complex patients.

## Consent

Written informed consent was obtained from the patient’s next-of-kin for publication of this case report and any accompanying images. A copy of the written consent is available for review by the Editor-in Chief of this journal.

## Abbreviations

AHI: apnea-hypopnea index; EDS: excessive daytime sleepiness; EtCO2: end-tidal carbon dioxide; GH: growth hormone; MSLT: multiple sleep latency test; NREM: non-rapid eye movement; PSG: polysomnography; PWS: Prader-Willi syndrome; REM: rapid eye movement; SDB: sleep-disordered breathing; SOREM: sleep-onset REM; SpO2: average oxygen saturation; TST: total sleep time.

## Competing interests

The authors declare that they have no competing interests.

## Authors’ contributions

All authors have made substantive intellectual contributions to this paper. All authors have contributed to the concept and design of the case report. RC, MBW and AMH have diagnosed and/or treated the patient. SVW, JLF, DR and AMH drafted the manuscript, and all authors have critically revised the manuscript for important intellectual content. All authors read and approved the final manuscript.
